# Evaluation of spatial-temporal variation performance of ERA5 precipitation data in China

**DOI:** 10.1038/s41598-021-97432-y

**Published:** 2021-09-09

**Authors:** Donglai Jiao, Nannan Xu, Fan Yang, Ke Xu

**Affiliations:** 1 School of Geographic and Biologic Information, Nanjing University of Posts and Telecommunications, Nanjing, 210023 Jiangsu Province China; 2 Department of Communication and Information Technology, Nanjing University of Posts and Telecommunications, Nanjing, 210023 Jiangsu Province China

**Keywords:** Atmospheric science, Hydrology

## Abstract

ERA5 is the latest fifth-generation reanalysis global atmosphere dataset from the European Centre for Medium-Range Weather Forecasts, replacing ERA-Interim as the next generation of representative satellite-observational data on the global scale. ERA5 data have been evaluated and applied in different regions, but the performances are inconsistent. Meanwhile, there are few precise evaluations of ERA5 precipitation data over long time series have been performed in Chinese mainland. This study evaluates the temporal-spatial performance of ERA5 precipitation data from 1979 to 2018 based on gridded-ground meteorological station observational data across China. The results showed that ERA5 data could capture the annual and seasonal patterns of observed precipitation in China well, with correlation coefficient values ranging from 0.796 to 0.945, but ERA5 slightly overestimated precipitation in the summer. Nonetheless, the results also showed that the accuracy of the precipitation products was strongly correlated with topographic distribution and climatic divisions. The performance of ERA5 shows spatial inherently across China that the highest correlation coefficient values locate in eastern, Northwestern and North China and the lowest biases locate in Southeast China. This study provides a reliable data assessment of the ERA5 data and precipitation trend analyses in China. The results provide accuracy references for the further use of precipitation satellite data for hydrological calculations and climate numerical simulations.

## Introduction

As one of the key types of measurements of the Earth's climate system and hydrological cycle, accurate precipitation measurements are vital to predicting weather, observing ecological changes, predicting droughts and floods, etc.^1–3^. Traditionally, precipitation data have been measured based on ground-based meteorological instruments and rain-gauge observations at fixed points. However, the spatial resolution of precipitation data obtained from ground-based meteorological stations is poor and susceptible to regional climatic influences, and the accuracy of the spatial distribution information of precipitation data is also insufficient^4^. With the development of remote sensing and computer technology, observing precipitation by satellite is becoming more advanced. Problems with uneven distributions of meteorological sites and radar signal interference can be avoided using satellites to observe precipitation^5^. Over the past decades, climate reanalysis data have been widely used in many fields, such as precipitation forecasting^6^, temperature prediction^7^, soil moisture monitoring^8^, ocean monitoring^9^, and environmental protection^10^, all of which use the laws of physics to combine model data with observations from around the world into a complete global dataset. These data with high spatial-temporal resolution can effectively compensate for the lack of direct ground-based precipitation observations.

The fifth generation of global climate reanalysis data, ERA5, published by the European Centre for Medium-Range Weather Forecasts (ECMWF), combines vast amounts of historical observations into global estimates using advanced modeling and data assimilation systems. Although there are several groups produce global atmospheric reanalysis and the most recent products are the MERRA-2 reanalysis^11^, JRA-55^12^ and CFSR (version 2)^13^, ERA5 have been shown to be the best or amongst the best performing reanalysis products by many studies^14–16^. ERA5 has much higher spatial and temporal resolution than ERA-Interim, providing quality precipitation data over space and time with a much improved troposphere and representation of tropical cyclones^17^. Compared to ERA-Interim, ERA5 also provides an enhanced number of output parameters. The move from ERA-Interim to ERA5 represents an increase in overall quality and the level of detail, which has superseded the ERA-Interim reanalysis data^18^. For example, Beck et al. (2019) found that ERA5 precipitation data provide significant improvement over ERA-Interim precipitation data at daily time steps against radar and precipitation gauge observations across the conterminous United States^19^. Hersbach et al. (2020) found that in comparison to ERA-Interim, the new reanalysis provides better precipitation data at a much higher spatial resolution on the global scale^20^. The ERA5 dataset is a valuable resource for climate change and scientists in the field of hydrological modeling and beyond. Additionally, some studies have documented the increased accuracy of ERA-5 over that of the ERA-Interim in matching observations for several variables, regions and periods. For example, Wang et al. (2019) estimated ERA5 and ERA-Interim precipitation data over the Arctic sea ice^21^. Zhang et al. (2019) analyzed the atmospheric precipitable water vapor of ERA5 over China^22^. Albergel et al. (2018) found that a land surface model forced by ERA5 could improve the simulation of evaporation, soil moisture, and river discharge over the continental United States^23^. Additionally, Nogueira (2020) evaluated ERA5 and ERA-Interim precipitation data using GPCP (Global Precipitation Climatology Project) as a reference at a global scale and found that it overestimated deep convection and moisture flux convergence over tropical oceans and land, leading to excessive rainfall^24^. Many studies of the precipitation performance of ERA-Interim have focused more on the spatial performance of reanalysis data at seasonal and annual scales^25,26^, which helps to enhance our understanding of data performance across all regions of China and over time.

ERA5 precipitation data has a huge improvement over ERA-Interim. Although ERA5 precipitation data have been widely evaluated, the performances are inconsistent in different regions^21–24^. Few researchers have evaluated the comprehensive performance of the data over a long time series in Chinese mainland^16^. Meanwhile, China has complex topography and pronounced climatic heterogeneity, which can affect precipitation amounts significantly. Therefore, the superiority of ERA5 precipitation data over China should be comprehensively analyzed. This study estimates the performance of ERA5 precipitation data from 1979 to 2018 based on gridded observational data (CN05.1) to compensate for the missing accuracy assessment of ERA5 precipitation data in Chinese mainland. The purposes of this study are to (1) evaluate the performance of ERA5 precipitation data against the observed data in China and (2) analyze the annual and seasonal trends and spatial patterns of ERA5 precipitation reanalysis data in China. The results are of great importance for the further rational use of reanalysis data and information on climate change and the hydrological cycle in China. The subsequent sections address the following: Sect. 2 introduces the dataset used in this study and the study area and methods. Section 3 focuses on the precision assessment results. Section 4 discusses factors that may influence the results. Finally, Sect. 5 concludes the paper.

## Materials and methods

### Study area

**Figure 1:**
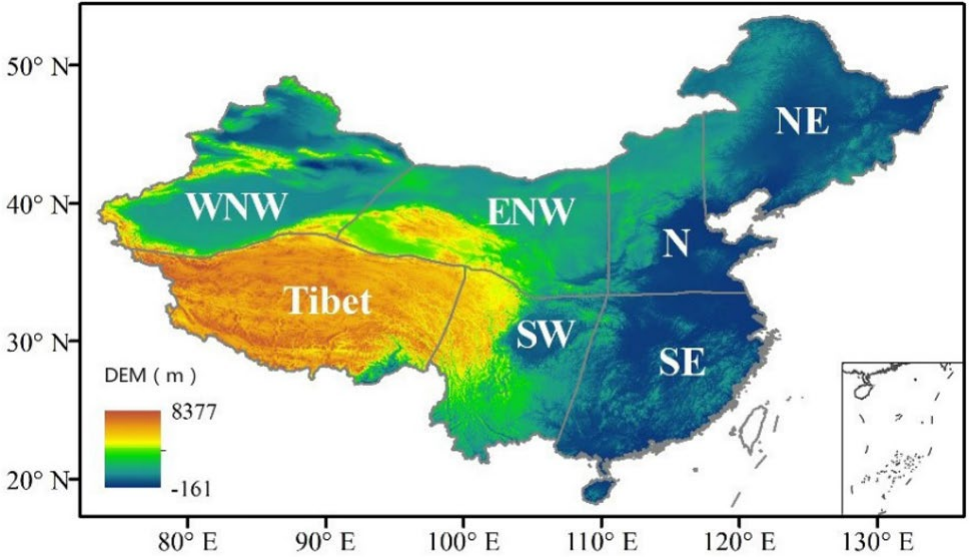
Elevation map and seven climatic divisions of China (ArcGIS Desktop. 10.0. ESRI, California, US. https://desktop.arcgis.com/zh-cn).

China locates in eastern Asia and has a complex topography, variable climate, and diverse terrestrial ecosystems. The precipitation shows significant spatial heterogeneities, decreasing from the southeastern coast to the northwestern interior. The southern region is affected by a tropical and subtropical monsoon climate, which is generally hot and rainy in summer and mild and wet in winter. The northern region is mostly affected by temperate monsoons and a temperate continental climate, with hot and rainy summers and cold and dry winters^27,28^. For analyzing the spatial heterogeneity characteristics of precipitation and the performance of ERA5, this study divides Chinese mainland into seven climatic zones according to previous studies of Wang et al. and Yang et al.^29,30^. The seven climatic zones are northeastern China (NE), northern China (N), southeastern China (SE), eastern northwestern China (ENW), southwestern China (SW), western northwestern China (WNW) and the Tibetan Plateau (Tibet) (Fig. 1).

### Datasets

The European Centre for Medium-Range Weather Forecasts (ECMWF), an international organization supported by 34 countries, is the world's leading international weather forecasting research and operations organization. ERA5 is the latest fifth-generation reanalysis of the ECMWF, which is expected to include detailed meteorological reanalysis data from 1950 onwards by 2020, completely replacing the previous ERA-Interim. ERA5 is a large upgrade over ERA-Interim with higher spatial-temporal resolution, obtaining for the hourly estimates of atmospheric variables at a horizontal resolution of 31 km, with a total of 137 mode layers of 0.01 hPa (approximately 80 km from the surface). ERA5 uses more historical observations, especially satellite data, in advanced data assimilation and modeling systems to estimate more accurate atmospheric conditions^31^. ERA5 uses a pooled reanalysis product consisting of 10 members with a temporal resolution of 3 hours and a spatial resolution of 31 km to assess atmospheric uncertainty. This new feature is based on the data assimilation aggregation (EDA) system developed by ECMWF and can explain errors in observation and prediction models, giving users more confidence when analyzing atmospheric parameters at different times and places. This study uses daily ERA5 precipitation data on regular latitude-longitude grids at 0.25° × 0.25° from 1979 to 2018.

The gridded observational precipitation data (CN05.1) developed by Wu et al. (2013)^32^ is used to evaluate the performance of ERA5 data. CN05.1 is produced based on observations from more than 2400 stations in China, using the thin-plate spline interpolation method with longitude and latitude as the thin-plate spline function and elevation as a covariate to interpolate the site data^33^. The data quality control uses a homogeneity analysis of the time series data and eliminates data with large deviations from historical records or surrounding sites. These observed data are validated in earlier studies^34,35^. The spatial distribution of elevation in Chinese mainland is based on NASA Shuttle Radar Topographic Mission (SRTM) Digital Elevation Model (DEM) data (Fig. 1).

### Statistical methods

This study estimates the temporal-spatial performance of ERA5 precipitation data using the validation statistical indices of the relative bias (Bias), correlation coefficient (CC), root-mean square error (RMSE), and standard deviation (SD) in seasonal and annual scale. In this study, Bias indicates the size of the deviation between the precipitation data from ERA5 and the observed precipitation data. The relative Bias can be calculated by Eq. (1):

1 $${\text{Bias}} = \frac{1}{{n*\overline{{X_{OBS} }} }}\mathop \sum \limits_{i = 1}^{n} \left( {X_{{ERA5_{i} }} - X_{{OBS_{i} }} } \right)*100\%$$

where the range of deviation is −∞~+∞ (the closer the deviation is to 0, the more accurate the data is) and Bias values >0 indicate overestimation, and <0 indicate underestimation.

The correlation coefficient (CC) reflects the degree of linear correlation between the ERA5 precipitation data and observed precipitation data (Eq. (2)).

2 $$CC = \frac{{\mathop \sum \nolimits_{i = 1}^{n} \left( {X_{{ERA5_{i} }} - \overline{X}_{ERA5} } \right)\left( {X_{{OBS_{i} }} - \overline{X}_{OBS} } \right)}}{{\sqrt {\mathop \sum \nolimits_{i = 1}^{n} \left( {X_{{ERA5_{i} }} - \overline{X}_{ERA5} } \right)^{2} } \sqrt {\mathop \sum \nolimits_{i = 1}^{n} \left( {X_{{OBS_{i} }} - \overline{X}_{OBS} } \right)^{2} } }}$$

where the range of CC is −1 to 1, completely correlated is 1 and completely uncorrelated is −1.

The RMSE reflects the overall level of error between the ERA5 precipitation data and the observed precipitation data, which can be interpreted as stable.

3 $$RMSE = \sqrt {\frac{1}{n}\mathop \sum \limits_{i = 1}^{n} \left( {X_{{ERA5_{i} }} - X_{{OBS_{i} }} } \right)^{2} }$$

where the range of RMSE is 0 to ∞, the smaller the value the smaller the overall deviation. For the above equations, $X_{{ERA5_{i} }}$ represents the precipitation data of ERA5, ${\text{X}}_{{{\text{OBS}}_{{\text{i}}} }}$ represents the observed precipitation, and $X_{ERA5}$ and $X_{OBS}$ represents average of precipitation for ERA5 and observation, respectively.

The SD can visually show and compare the “closeness” between the ERA5 and observation precipitation data^36^.

4 $$SD = \sqrt {\frac{1}{n}\mathop \sum \limits_{i = 1}^{n} \left( {X_{i} - \overline{X}} \right)^{2} }$$

where *n* is the number of samples, *i* is the i^th^ grid, *X*_*i*_ is the precipitation estimated from ERA5 and observation, respectively. $\overline{X}$ is the average precipitation of the samples.

The line trend (or slope) of the precipitation at the spatial scale is solved by the least-square method^37^.

5 $$Slope = \frac{{n\sum {X_{i} Y_{i} - } \sum {X_{i} Y_{i} } }}{{n\sum {X_{i}^{2} - \left( {\sum {X_{i} } } \right)}^{2} }}$$

where *X*_*i*_ is the ith year, *Y*_*i*_ represents the precipitation in year i, and n is the total number of years.

The Mann–Kendall test can examine the trend of the series by making the following assumptions about the time series:

1. H0 hypothesis. It is assumed that the data in the series are independent identically distributed random samples, i.e., there is no significant trend.
2. H1 hypothesis. It is assumed that there is an upward or downward monotonic trend in the series. Under the H0 hypothesis, the test statistic S is defined as:

6 $$S = \mathop \sum \limits_{i = 1}^{n - 1} \mathop \sum \limits_{j = i + 1}^{n} sgn\left( {x_{j} - x_{i} } \right)$$

where *sgn* is the symbolic function and $$sgn = \left\{ {\begin{array}{*{20}l} {1,} \hfill & {\theta > 0} \hfill \\ {0,} \hfill & {\theta = 0} \hfill \\ { - 1,} \hfill & {\theta < 0} \hfill \\ \end{array} } \right.$$. In Eq. (6), when n ≥ 10, the statistic *S* approximately obeys a normal distribution. *S* is normalized to obtain Z, and the significance test is performed using the statistical test value *Z* with the following formula:

7 $$Z = \left\{ {\begin{array}{*{20}l} {\left( {S - 1} \right)/\sqrt {var\left( S \right)} ,} \hfill & {S > 0} \hfill \\ {0,} \hfill & {S = 0} \hfill \\ {\left( {S + 1} \right)/\sqrt {var\left( S \right)} ,} \hfill & {S < 0} \hfill \\ \end{array} } \right.$$

8 $$var\left( S \right) = \left( {n\left( {n - 1} \right)\left( {2n + 5} \right) - \mathop \sum \limits_{i = 1}^{m} t_{i} \left( {t_{i} - 1} \right)\left( {2t_{i} + 5} \right)} \right)/18$$

where *n* is the number of data in the sequence; m is the number of knots (recurring data groups) in the sequence; $t_{i}$ is the width of the knot (the number of repeated data in the ith set of repeated data groups).

Using the bilateral trend test, the $H_{0}$ hypothesis is accepted when $\left| Z \right| \le Z_{1 - \alpha /2}$ at a given significant level* α*, i.e., the trend is not significant; otherwise, the $H_{1}$ hypothesis is accepted, i.e., $Z > Z_{1 - \alpha /2}$ indicates a significant upward trend of the series, and $Z < - Z_{1 - \alpha /2}$ indicates a significant downward trend of the series.

The spatial distribution of precipitation is clearly affected by the topography. Therefore, this study calculates the CC and Bias of the EAR5 and the observed precipitation data based on four elevation categories: plain areas below 1000 m, medium and high altitude areas from 1000 to 2000 m, higher altitude areas from 2000 to 3500 m, and ultra-high altitude areas above 3500 m.

In addition, the empirical orthogonal function (EOF) is also used to check the consistency of both datasets. In this method, the sampling error method are used to select the leading eigenvectors of EOF analysis and corresponding principal components. Meanwhile, for easier comparison, both ERA5 and observed data are forced to have the same leading modes, which are decided by the minimum number of leading modes between ERA5 and observed data.

## Results and discussion

### Results

**Figure 2:**
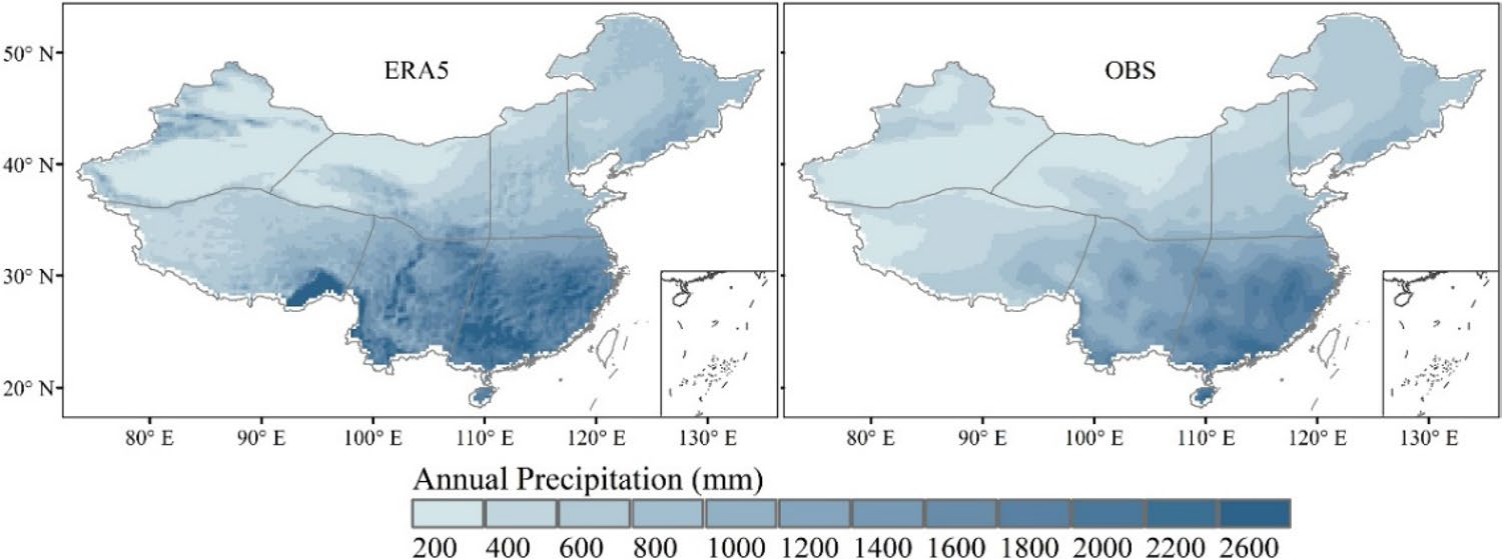
Spatial patterns of annual mean precipitation of ERA5 (right panel) and observation (OBS, left panel) from 1979 to 2018 (ArcGIS Desktop. 10.0. ESRI, California, US. https://desktop.arcgis.com/zh-cn).

The annual mean precipitation of ERA5 shows similar spatial distribution with that of the observation from 1979 to 2018 (Fig. 2). Figure 2 shows that the annual mean precipitation pattern gradually decreases from the southeast (> 2000 mm) to the northwest (< 200 mm) of China. This is mainly due to Chinese mainland is located in the typical Asian monsoon region where the monsoonal circulation significantly affects the spatial distribution of precipitation. However, the annual mean precipitation data of EAR5 varies from 13 to 2988 mm, which has a wider range than that of the observation. Meanwhile ERA5 overestimates the annual precipitation in the southeastern part of the Tibet Plateau compared to the observation. It is maybe caused by the sparse weather stations in northwest China and the limited observation cannot capture the precipitation pattern with enough details.

**Figure 3:**
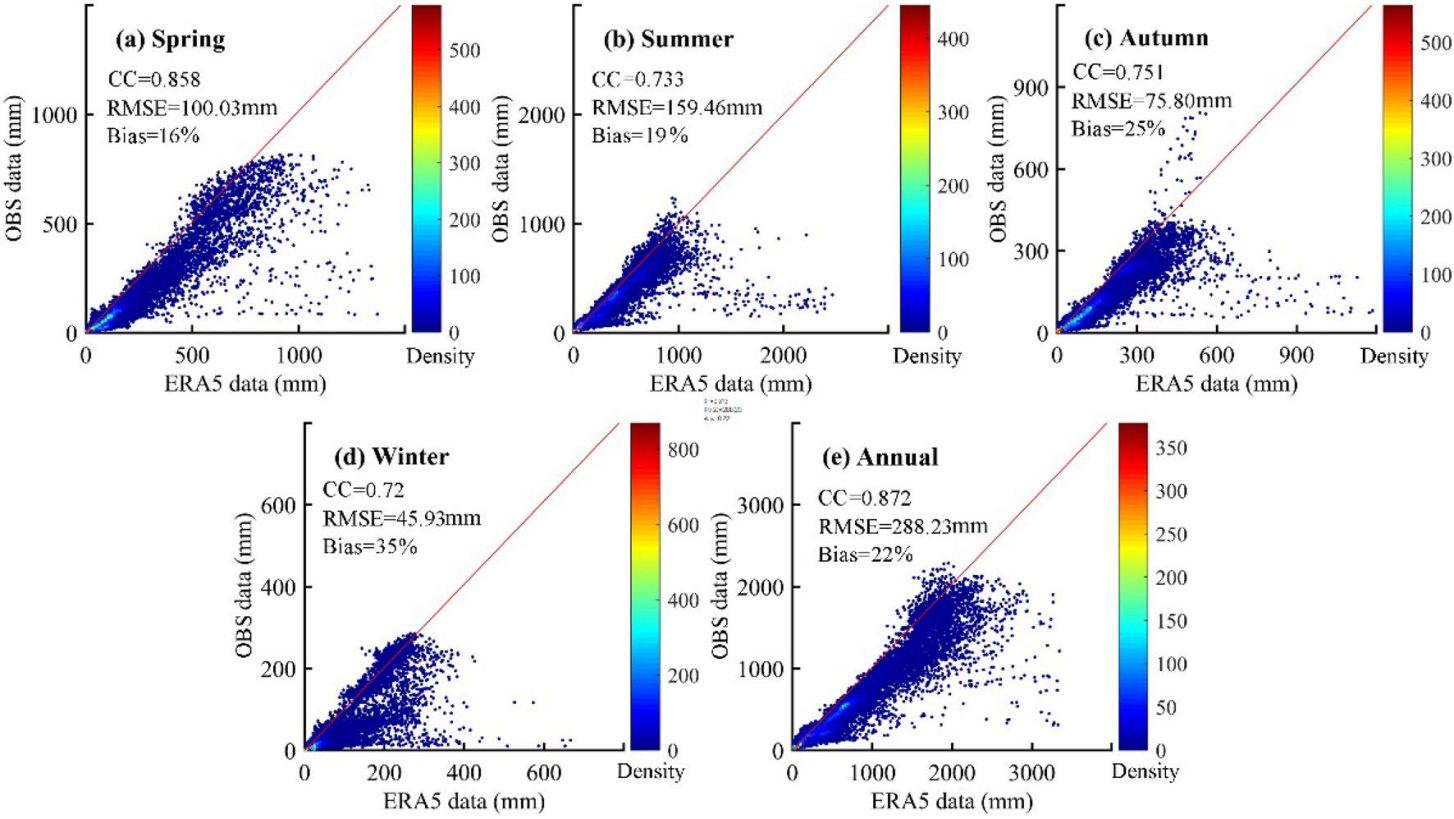
Seasonal (**a**–**d**) and annual (**e**) density scatter plots of observation (OBS) and ERA5 precipitation data in China from 1979 to 2018. The 1:1 line of perfect agreement (red line) and the colour represents the density of the grids. Note: correlation coefficient (CC), root-mean square error (RMSE), and relative bias (Bias).

Figure 3 presents the seasonal and annual Bias, RMSE, CC, and density scatter plot for the ERA5 and observed precipitation data in Chinese mainland. The 40-year annual average precipitation from the ERA5 and observations data is highly correlated with the corresponding CC, RMSE and Bias values at 0.872, 288.23mm, and 22%, respectively. As can be seen in Fig. 3, the precipitation data of ERA5 are slightly higher than the ground observations, both on the annual and seasonal scale, but at the same time, the CCs all above 0.7. This indicates that there is a slight overestimation of the satellite precipitation data, but overall, the accuracy is high. For seasonal total precipitation, good performance is observed for spring precipitation, with a coefficient of determination CC of 0.858, a Bias of 16%, and an RMSE of 100mm. Winter precipitation has the lowest RMSE, with scare precipitation in this season. In contrast, the ERA5 data has a higher RMSE value (more than 150mm) and lower Bias (19%) in the summer, which indicates that the ERA5 data has a lower deviation from the observation data but a relatively higher error than that in other seasons. In general, the seasonal-scale precipitation data of the ERA5 has a good linear relationship with the observation, with slight overestimation in different seasons but with very small errors and high overall accuracy.

To better understand the differences between the zones, Table 1 lists the quantiles (5%, 50% and 95%) of statistical indicators (CC, RMSE, and Bias) at a grid scale in the seven study zones for the seasonal ERA5 precipitation data against to the observation. It shows that almost all divisions have higher CC and lower RMSE in the spring than in the summer, except for the SE region. In addition, the Bias in the summer in all seven zones is lower compared to that in the other three seasons. This indicates that ERA5 can adequately determine extreme precipitation. The highest CC occurs in the ENW region, but the lowest RMSE and Bias are in the N and NE regions, respectively. This maybe relates to their lower elevation and temperate continental climate, and the fact that weather stations are also more abundant in these areas, providing more accurate station data. The Tibetan Plateau region has the largest deviation regardless of the season, which means that ERA5 reanalysis data are weak for monitoring precipitation in the Tibetan region, and special attention should be paid to this region when applying the data. Generally, ERA5 precipitation data has reliability and high precision in ENW, N, and NE zones for all seasons, while higher Bias and RMSE are located in Tibet (Table 1). At the same time, Tibet has the lowest CC in the summer and winter.

**Table 1:** 5%, 50%, and 95% quantiles of the CC (), Bias (%), RMSE (mm/month) for annual and seasonal precipitation data between ERA5 and observation in seven zones of China.

Zone	Quantiles (%)	Spring			Summer			Autumn			Winter		
		CC	Bias	RMSE	CC	Bias	RMSE	CC	Bias	RMSE	CC	Bias	RMSE
WNW	5	*0.26*	19.59	*4.95*	0.36	−5.81	*12.33*	0.29	8.99	*4.48*	0.33	25.76	*1.79*
	50	0.66	43.17	17.34	0.62	6.95	29.77	0.64	30.22	14.10	0.62	68.72	7.47
	95	0.87	76.56	124.29	0.81	22.72	264.14	0.81	48.29	94.82	0.85	121.52	30.69
ENW	5	0.45	−45.75	5.75	0.28	−57.59	17.48	0.58	−52.89	5.09	0.25	−38.00	1.88
	50	0.74	20.56	22.92	0.68	−12.68	46.77	0.77	18.95	24.07	0.57	22.94	8.14
	95	0.91	**339.05**	86.08	0.84	**237.82**	174.18	0.86	254.02	116.87	0.83	263.60	46.02
N	5	0.68	−20.16	16.38	0.60	−35.85	29.07	0.67	−27.36	15.32	0.42	*6.79*	5.19
	50	0.81	36.33	35.40	0.75	11.31	87.21	0.83	30.65	35.27	0.81	92.22	11.57
	95	0.90	117.99	55.39	0.86	69.34	147.38	**0.93**	112.02	58.79	0.94	305.51	20.95
NE	5	0.63	9.41	21.48	0.67	−7.02	37.15	0.67	*2.96*	18.14	0.57	14.50	5.68
	50	0.84	30.78	39.04	0.83	8.98	58.83	0.85	18.90	35.08	0.83	58.86	9.78
	95	**0.92**	69.30	72.75	**0.90**	34.11	126.39	0.92	49.33	57.31	0.91	122.93	29.57
Tibet	5	0.00	*2.48*	13.07	*0.08*	6.52	40.36	*0.03*	−25.29	16.36	*0.13*	−25.28	5.25
	50	0.50	126.04	51.03	0.50	48.97	109.70	0.55	110.63	44.94	0.46	212.00	18.69
	95	0.78	321.73	326.06	0.84	201.17	**450.18**	0.78	**839.43**	**271.69**	0.70	339.14	**180.58**
SW	5	0.28	7.54	39.70	0.32	−*4.39*	67.88	0.45	28.15	44.87	0.36	28.71	21.11
	50	0.64	46.06	105.01	0.63	20.57	153.80	0.71	146.62	97.74	0.69	157.73	59.15
	95	0.82	168.67	276.22	0.82	68.83	412.69	0.85	601.52	233.66	0.86	**601.44**	170.02
SE	5	0.37	−7.52	51.99	0.61	−10.26	99.57	0.72	−7.04	41.95	0.65	−14.34	20.76
	50	0.66	16.56	139.11	0.76	14.35	161.20	0.85	8.98	64.10	0.84	8.23	43.94
	95	0.84	64.40	**368.13**	0.85	39.29	284.99	**0.93**	40.43	124.56	**0.95**	108.98	143.57

The maximum and minimum values of each column are shown with bold and italics style, respectively, except for the Bias, whose minimum value is shown as the absolute minimum value.

**Figure 4:**
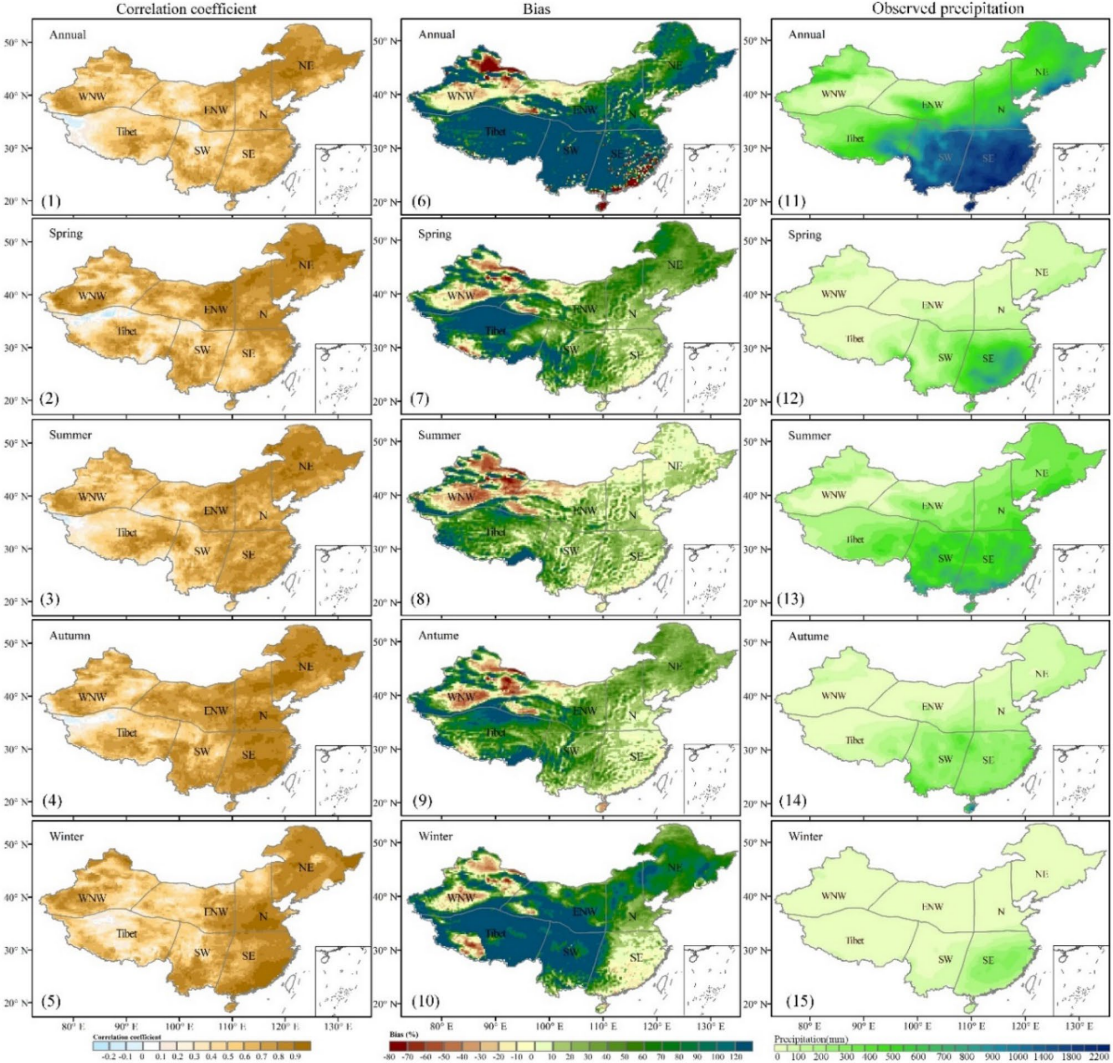
The correlation coefficient (CC) (left panel, (1)–(5)) and Bias (middle panel, (6)–(10)) between the ERA5 and observed precipitation data (right panel, (11)–(15)) during 1979–2018 in Chinese mainland (ArcGIS Desktop. 10.0. ESRI, California, US. https://desktop.arcgis.com/zh-cn).

To further estimate the performance of the ERA5 data in terms of the temporal and spatial patterns of the observed precipitation data in Chinese mainland, Fig. 4 presents the spatial maps of the correlation coefficient (Fig. 4(1)–(5)) and Bias (Fig. 4(6)–(10)) between the ERA5 and observations data. It is evident from the annual scale that the correlation coefficients are higher in SE, N, and NE China than in the other regions, and the value of CC is generally above 0.75, suggesting high reliability of the ERA5 precipitation data in these regions. The ERA5 data has a significantly higher CC values in eastern China than in western China. At the same time, the higher spring CC in all regions contributes more to the annual correlation coefficients, except in southeastern China. However, the CC values in most parts of the Tibetan Plateau and northwestern and southwestern China are below 0.6 in all seasons, indicating that the ERA5 data are in better agreement with the observational data at lower elevations in eastern China (CC>0.9) than at higher altitudes in the western region (CC<0.6).

The ERA5 data generally has a smaller Bias in southeastern China, mostly below ±20% (Fig. 4(5)–(10)). In contrast, the Bias in Tibet is mostly positive and generally higher than 100%. This indicates that the ERA5 precipitation data seems to greatly overestimate precipitation in this region. At the seasonal scale, the greatest seasonal differences in precipitation are observed between southwestern China and the Tibetan Plateau region (from 258 ± 20 mm in the summer to 40.2 ± 8.5 mm in the winter), followed by the southeastern region, with a 210.4 ± 98.5 mm difference in total annual precipitation; all other regions have differences of less than 50 mm, with summer and spring contributing more than the other seasons to the interannual differences (Fig. 4(12)–(13)). It indicates the ERA5 precipitation data can capture the spatial pattern of the observed seasonal precipitation; however, the ERA5 overestimates the seasonal precipitation in Tibet and SW regions, especially in spring and winter (Fig. 4(7) and (10)).

**Figure 5:**
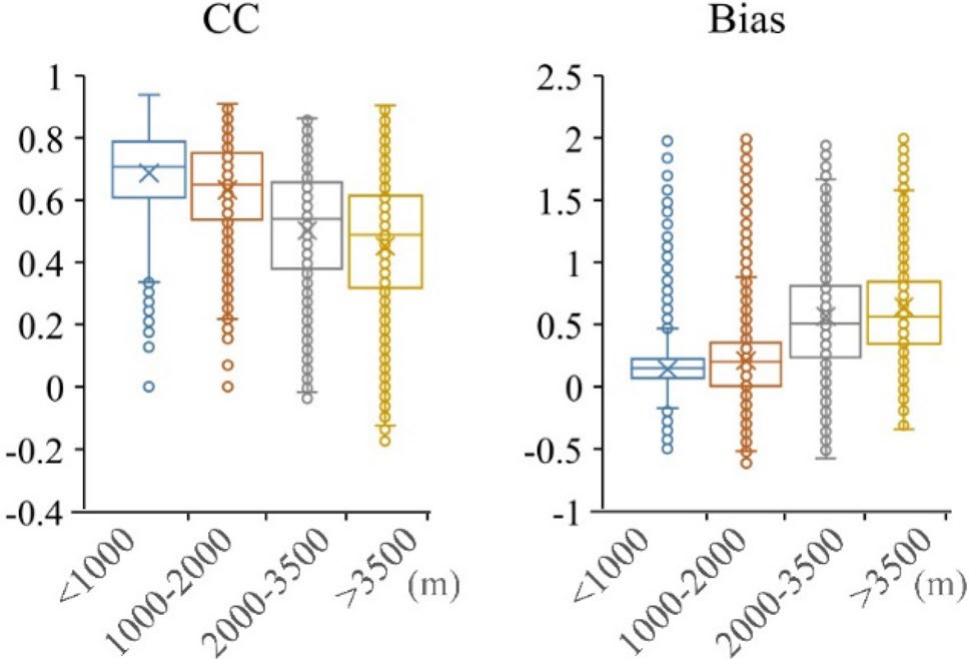
Box plots of CC and Bias between the ERA5 and observed annual precipitation data with different elevation during 1979–2018 in Chinese mainland.

Figure 5 presents the performance of ERA5 precipitation data on different terrain (below 1000 m, medium and high altitude areas from 1000 m to 2000 m, higher altitude areas from 2000 m to 3500 m, and ultra-high altitude areas above 3500 m). It shows that the CC values decrease with increasing altitude, with the highest correlation in the plains and the lowest correlation at high altitudes in the Tibetan region. It indicates that the ERA5 precipitation data provides poor estimates of precipitation at high altitudes. Meanwhile, the Bias values increase with altitude and are all positively biased, indicating that the ERA5 precipitation data are overestimated at most pixels.

**Figure 6:**
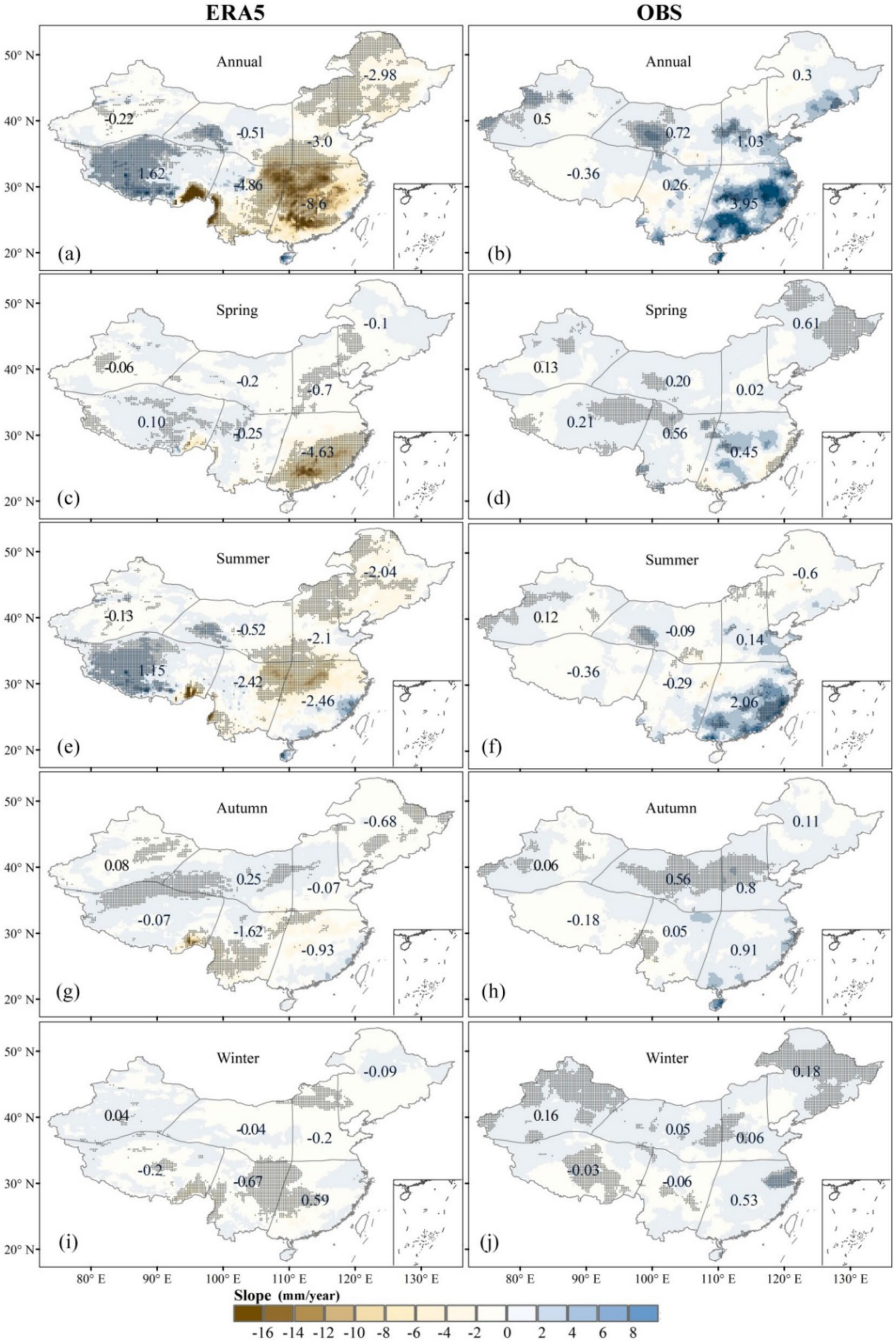
Spatial patterns in annual and seasonal slope variations (1979–2018 average) in Chinese mainland. The number is the average slope of each division. Note: The black points indicate trends significant at 95% confidence level by Mann–Kendall test (ArcGIS Desktop. 10.0. ESRI, California, US. https://desktop.arcgis.com/zh-cn).

Figure 6 presents the change trend in annual and seasonal precipitation between the ERA5 and observations data. EAR5 can generally catch the seasonal changing trend pattern of the observation. However, the spatial patterns of changing trends show more inconsistencies between ERA5 and the observation in annual and seasonal scale. The ERA5 annual precipitation shows a clear downward trend in SE (−8.6 mm/year), N (−3.0 mm/year), and NE (−2.98 mm/year) of China, but observed precipitation shows an upward trend in these regions (3.95, 1.03, and 0.3 mm/year in the SE, N, and NE, respectively).

**Figure 7:**
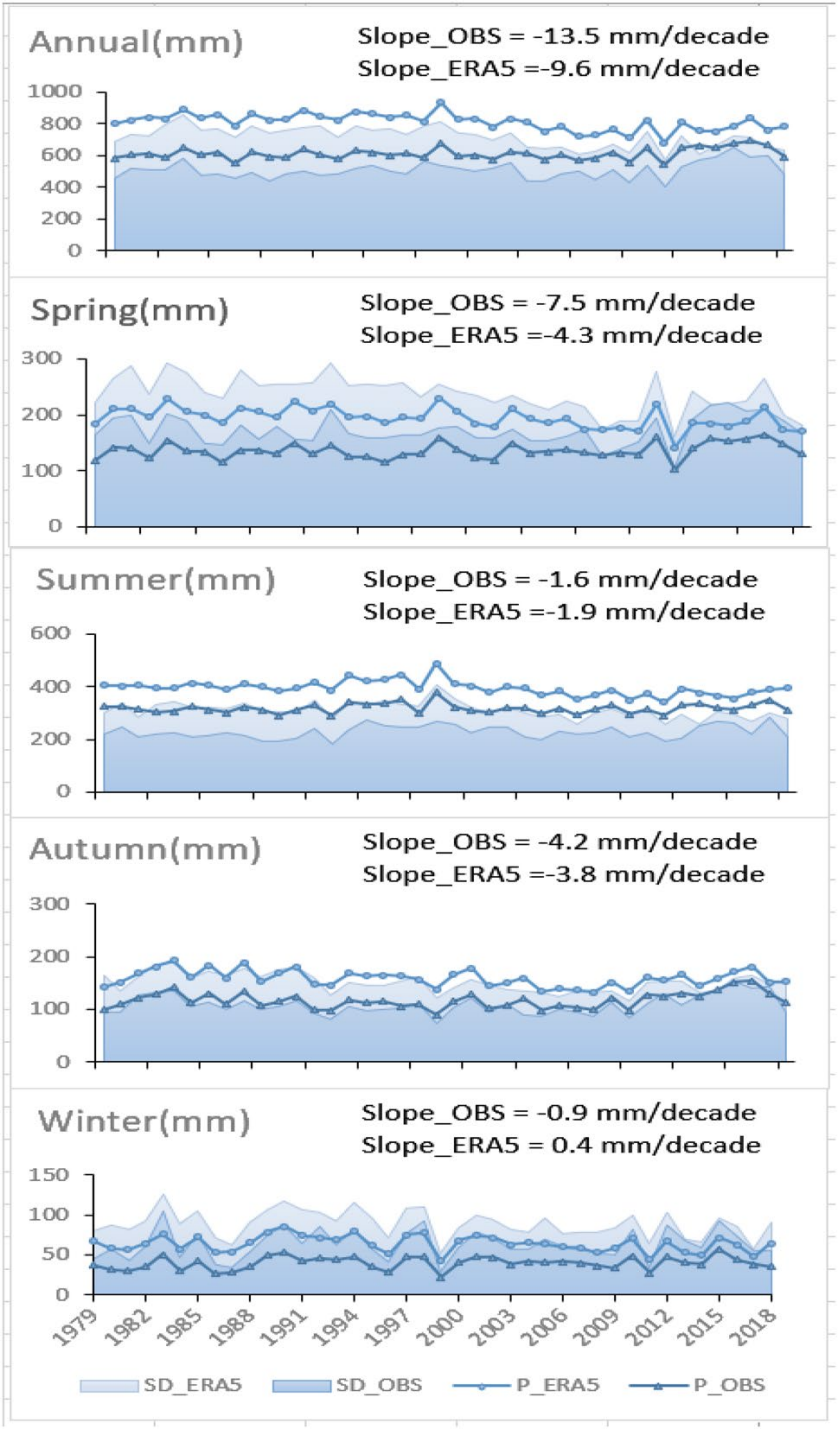
Graph of trends in annual and seasonal precipitation in Chinese mainland during 1979–2018. The dark blue and light blue lines are the spatial mean observation and ERA5 precipitation data, respectively. The light blue and dark blue zones are the spatial averaged SD of the ERA5 and observed precipitation data, respectively. (The slope values in the graph refer to the slope values of precipitation over these 40 years.).

Figure 7 presents the spatial averaged SD and its changing trends for the ERA5 and observation data at the annual and seasonal scales. The SDs of the observation and ERA5 data show consistent trends at both the annual and seasonal scales, with the largest SDs in the summer and the smallest in the winter, indicating that precipitation has temporal variability. It should be noted that the ERA5 precipitation data typically displays a higher SD value than the observed precipitation data, varying from 88.3 mm in the winter to 309.5 mm in the summer. For 1979–2018, the trends of the SD values for the ERA5 and observation data at the annual scale are −12.25 mm and −8.64 mm per decade, respectively, indicating a decrease in the temporal variability in annual precipitation. Similar decreasing trends are observed in spring, autumn, and winter. The SD of summer precipitation shows a slight increasing trend in both the ERA5 and observation by 2.03 mm and 2.38 mm per decade, respectively.

As also can be seen in Fig. 7 that the annual and seasonal precipitation trends are basically similar during the period 1979–2018, both showing a decreasing trend, with only a slight upward trend in winter, rising by 0.4 mm per decade. The contributions of spring and autumn to the declining trend in annual precipitation were slightly larger than those of summer and winter, with changes of more than 3 mm per decade in spring and autumn and less than 2 mm per decade in summer and winter. The change in winter precipitation was the weakest, with 0.4 mm per decade for the observation data and 0.9 mm per decade for the ERA5 data. It is noteworthy that relative to the decreasing trend of 4.3 mm per decade in spring precipitation data from the observed data, the spring precipitation data from ERA5 decrease by 7.5 mm per decade and contribute the most to the decreasing trend in annual precipitation.

**Figure 8:**
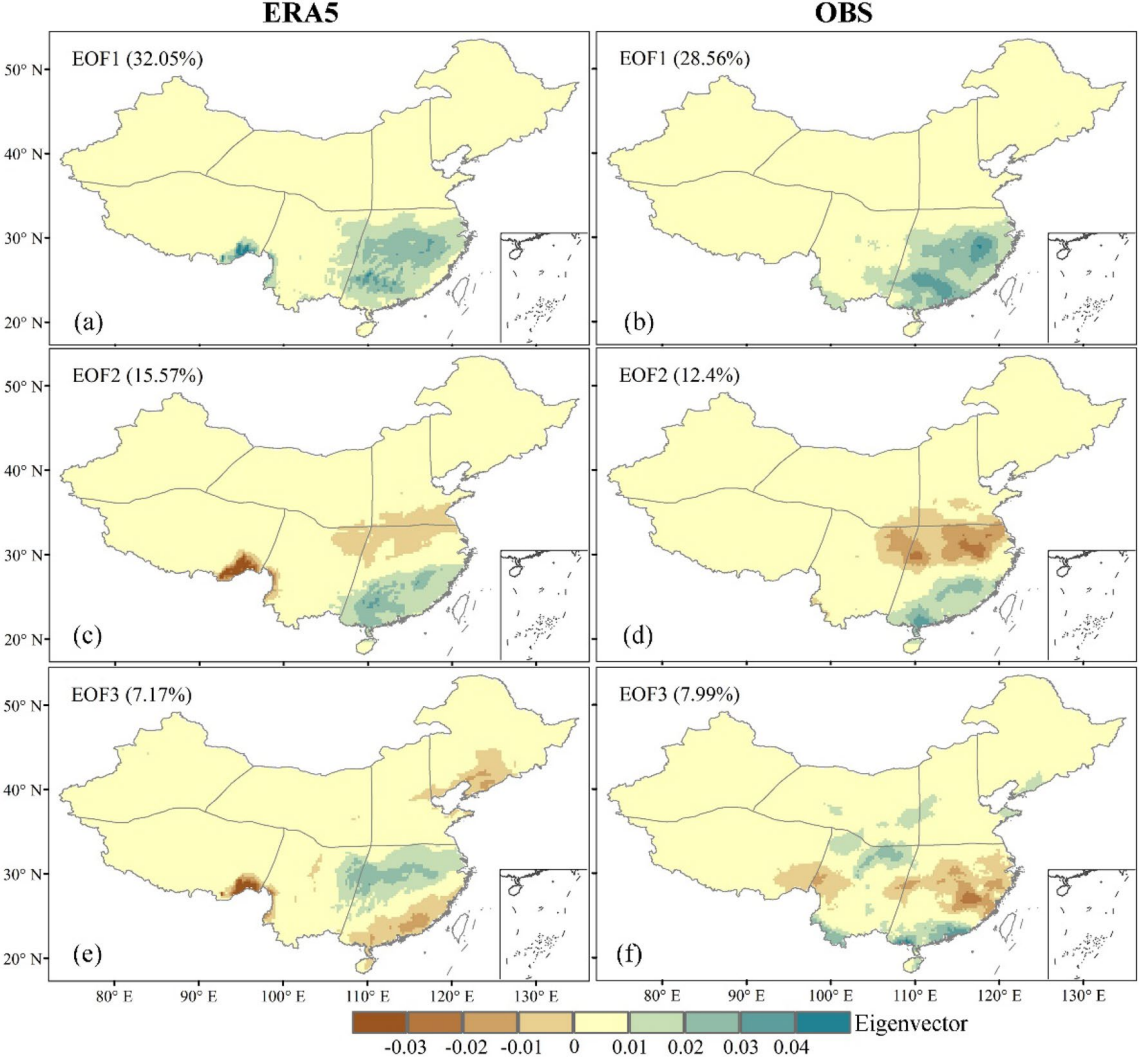
First three leading eigenvectors derived from the EOF analysis of ERA5 (left panel) and observed (right panel) annual precipitation over Chinese mainland from 1979 to 2018.All maps were prepared using ArcGIS Desktop 10.0 (ESRI, California, US. https://desktop.arcgis.com/zh-cn).

The top three leading EOF modes for the ERA5 and observed annual precipitation are presented in Fig. 8. The percentage of total variance explained by the top three main EOF modes exceeds 54% in the ERA5 data and 48% for the observed data. The first and second modes differ somewhat in the proportion of the total variance of the EOF modes corresponding to ERA5 and observed annual precipitation, while the third mode is similar (32.05% vs28.56% for EOF1. 12.4% vs 15.57% for EOF2. 7.99% vs. 7.17% for EOF3). Although EOF1 and EOF2 patterns for ERA5 annual precipitation are identical to those of the observation, they show significant differences in some areas of EOF3. The increasing trend of annual precipitation in almost all areas could be captures by EOF1 (positive values) of both ERA5 and observation, but ERA5 shows 10% more precipitation than observation on the southeast corner of the Tibetan Plateau.. For EOF2, the opposite region is also concentrated in the southeast corner region of the Tibetan Plateau, where observation shows negative values and ERA5 positive values. For EOF3, the annual precipitation of ERA5 decreases in the 25°N-30°N region with negative value, while the annual precipitation of observation increases with positive value. It indicates that It indicates that the spatial patters between the ERA5 and the observed precipitation are generally similar explained by the first and second EOF modes.

## Discussion

Satellite reanalysis of precipitation data are widely used in hydrology, meteorology and climatology and their applications. However, it is difficult to quantitatively estimate the accuracy of satellite reanalysis of precipitation data due to their complex variability at spatial and temporal scales. This study estimates the seasonal and annual temporal and spatial performance of the ERA5 precipitation data against observational data in China from 1979 to 2018. The ERA5 precipitation data can capture the temporal-spatial pattern of the observed precipitation in China, which can be used for hydrological and climatic studies in China. There is good agreement between the ERA5 precipitation data and observational data at lower elevations (below 1000 m) in eastern China, but not at higher elevations in western China. ERA5 precipitation data overestimates the spring and summer precipitation. The overestimate also occurs in the southeastern part of the Tibet Plateau for annual scale. The overestimation may be related to inverse algorithms used for satellite products and inaccurate estimates of solid precipitation^16^. Unlike previous studies on specific regions or seasons^38,39^, this study provides a deeper understanding of the continuous spatiotemporal performance of ERA5 precipitation data from different climate zones and temporal scales in Chinese mainland. We find that the annual and seasonal ERA5 precipitation performance varies spatially across the western, central, and eastern regions. These evaluations help to deepen our understanding of the uncertainty in the ERA5 precipitation data in different regions and seasons in China.

As a new generation of the ECMWF reanalysis data, ERA5 performs better than ERA-Interim in representing the precipitation variability with higher spatial and temporal resolution than ERA-Interim, providing quality precipitation data over space and time^18–21,24^. This study finds that the ERA5 precipitation at lower elevations is estimated more accurately and the trends in precipitation at annual and seasonal scales are more consistent with observations, probably because ERA5 has much higher spatial and temporal resolution than ERA-Interim, providing higher quality precipitation data in both space and time^25^. To obtain more meaningful climate variables, reanalysis systems often take into account ground pressure, 2 m air temperature, 2 m relative humidity and 10 m wind speed, all of which are observations considered within the reanalysis system that contribute to improved data quality^40^. However, we must consider that although the data assimilation approach can improve data accuracy by adding physically meaningful information from the predictive model, it is still subject to uncertainty^40^. For example, numerical simulations, assimilation schemes and errors in observation systems may affect the ability of ERA5 data to capture the actual climate. Therefore, some studies have shown that it is difficult to completely replace observational data information with reanalysis system information to reflect the true state of the atmosphere^41^. For example, Wang et al.^25^ showed in their study that ERA-Interim reanalysis data are not suitable for long-term climate trend calculations. In this paper, we also note that seasonal and interannual changes in the ERA5 data are not entirely consistent with changes in the observational data.

Usually, the stations are located in plains or mountain valleys, which mean that interpolations on the surrounding alpine grid points need to be revised in terms of topography. The dataset used in this study, CN05.1, was implemented using ANSPLIN software, and the resulting revised coefficient is a uniform value across the application area, which varies somewhat depending on the number of sites used. This dataset still has the uncertainties which can also influence the validation result, although this dataset has been quality controlled and estimated by other studies^32–35^. In the future, consideration could be given to interpolating the values separately in different regions after appropriate partitioning by climate characteristics. In addition, the use of reanalysis data to drive high-resolution regional climate models could be attempted and spatially and temporally varying terrain revision parameters could be analyzed in the simulation results and used to interpolate the observations. Therefore, we should also consider the uncertainty in the CN05.1 dataset when evaluating accuracy.

This study finds that the ERA5 precipitation data are in better agreement with the observational data at altitudes below 1000 m, with deviations close to 1% and a general CC more than 0.6. However, significant differences are observed in mountainous areas with an average elevation above 4000 m, especially on the Tibetan Plateau. This indicates that elevation-induced Bias may be the cause of uncertainty in the accuracy of the data, while the scarcity of meteorological stations at high altitudes may also lead to Bias in the generated gridded data. Therefore, when applied to Tibetan Plateau, the altitude correction and gridded data analysis should be taken into account^39,42–44^. Zhao et al. analyzed the relationship between terrain correction and reanalysis of surface temperature errors between National Centers for Environmental Prediction–National Center for Atmospheric Research (NCEP–NCAR) and ERA-40 using meteorological station data and found that the deviation is usually proportional to the increase in local elevation and terrain complexity^45^. Gao and Hao analyzed the difference between ERA-Interim elevation and observed station elevation and pointed out that elevation differences can affect the accuracy of the reanalysis data, especially in areas with higher elevations^46^.

## Conclusions

This study synthesizes and evaluates the performance of the ERA5 annual and seasonal precipitation data at different temporal scales and locations from 1979 to 2018 based on gridded observational data in China. The results show that the ERA5 precipitation data can capture the temporal-spatial patterns of the observed precipitation in China, with a generally high accuracy but slight overestimation of regional precipitation over Chinese mainland, especially in the summer. However, the trend variation of ERA5 precipitation data is dramatically different from the observed data in spatial distribution, both on annual and seasonal scales. The accuracy of the precipitation products is strongly correlated with the topographic distribution and climatic divisions. Furthermore, this study suggests that extra care should be taken to consider precipitation uncertainty at high altitudes when applying the ERA5 precipitation reanalysis data. These results help us to further understand the error sources, the rational application of the reanalysis products and the potential improvements for the next generation of products.

## Data Availability

ERA5 hourly data on single levels from 1979 to present are available from the ECMWF, please visit: https://cds.climate.copernicus.eu. The digital elevation data can be downloaded from the shuttle radar topography mission (SRTM) digital elevation model (https://eospso.gsfc.nasa.gov/missions/shuttle-radar-topography-mission).
